# The Prevalence and associated Factors for Liver Metastases, Development and Prognosis in newly diagnosed Epithelial Ovarian Cancer: A large Population-Based Study from the SEER Database

**DOI:** 10.7150/jca.40590

**Published:** 2020-06-08

**Authors:** Iftikhar Hussain, Jiaqin Xu, Kui Deng, Noor-ul-Amin, Ce Wang, Yue Huang, Shuang Li, Kang Li

**Affiliations:** Department of Epidemiology and Biostatistics, School of Public Health, Harbin Medical University, Harbin 150086, People's Republic of China.

**Keywords:** Liver Metastases, Epithelial Ovarian Cancer, SEER database, Survival, Risk factors, Prognostic factors

## Abstract

**Background:** Primary Epithelial Ovarian Cancer (EOC), a malignant gynecologic disease, is considered one of the leading causes of mortality in women. The development of Liver Metastases (LM) in women with primary ovarian cancer commonly results in a poorer prognosis. This retrospective population-based study aims to measure the prevalence, prognostic factors, and associated risk factors for epithelial ovarian cancer patients with liver metastases (EOCLM).

**Materials and Methods:** The current study cohort of patients based on the Surveillance, Epidemiology, and End Results (SEER) database identified with primary ovarian cancer between the years 2010 and 2016. A chi-square test was employed to compare Metastatic differences among demographic and clinical factors. Univariable and multivariable logistic regression analysis models were used to predict related prognostic factors for LM development. 7-year Kaplan-Meier curves were applied to compare the survival patterns of patients with and without LM. The Multivariable Cox regression model was used to estimate potential risk factors associated with LM related deaths.

**Results:** 33895 eligible primary EOC patients were identified. Among them 2635 (7.77%) patients were initially diagnosed with de novo LM, and 31260 (92.23%) without metastases disease to any site. Non-serous histology type; Malignant Brenner Carcinoma, NOS (OR 1.94; CI: 1.39-2.71; *P*<0.001), T3/T1 stage (OR 5.65; CI: 3.87-8.24; *P*<0.001), N1/N0 stage (OR 1.67; CI: 1.43-4.95; *P*<0.001), grade; G3/G1 (OR 2.16; CI: 1.29-3.59, *P*<0.001), and cancer antigen-125; Elevated/Normal (OR 1.79; CI: 1.19-2.69, *P*<0.001) were significantly associated with LM occurrence. The median survival of EOC patients with LM was 12.0 (95% CI: 11.0-14.0; *P*<0.001) months. Multivariable cox regression showed being unmarried (HR 1.16; CI: 1.04-1.30; *P*=0.001), non-serous histology types, Mucinous (HR 2.38; CI: 1.82-3.12; *P*<0.001), Clear cell (HR 1.83; CI: 1.32-2.55; *P*<0.001), Malignant Brenner Carcinoma, NOS (HR 1.44; CI:1.23-1.66; *P*<0.001), Carcinosarcoma NOS, (HR 1.44; CI: 1.11-1.88; *P*<0.001) and radiotherapy (HR 1.52; CI: 1.12-2.06; *P*<0.001), were positively related to death. Chemotherapy (HR 0.30; CI: 1.12-2.06; *P*<0.001) and surgery (HR 0.34; CI: 0.29-0.39; *P*<0.001) were related with reduced rate of death.

**Conclusion:** The retrospective cohort study showed that women with primary EOC had some high-risk factors associated with LM. LM can intensely decrease the survival of EOC patients. The findings of our research provided estimates for LM occurrence prediction and potential prognostic factors of EOC with de novo LM development. These findings can be useful for follow-up strategies, guidelines for screening, and treatment of EOCLM.

## Introduction

Epithelial Ovarian Cancer considered among the fatal gynecologic malignancies of the female reproductive system, with expected 22,530 new cases and 13,980 deaths to occur in 2019 in the US 1,2. Ovarian cancer accounts for 2.5% of all fatalities among women. Almost 60% of EOC patients diagnosed at a distant stage. The 5-year survival of EOC patients is still less than 47% 3. The lifetime risk of ovarian cancer incidence is 1.39%, and the lifetime risk of death is 1.04% 2. The majority of EOC patients diagnosed with advanced stages (III and IV) due to no practical screening tests and symptoms are distinct. About 12-33% of EOC patients will be diagnosed with FIGO stage IV, de novo Metastases disease at initial diagnosis 4. Liver Metastases found in up to 50% of patients dying of EOC 5,6. Recent studies noted liver Metastases reported as the most common cause of stage-IV disease in EOC patients 4,7. Hepatic resection of patients with primary ovarian cancer with liver metastases could benefit in terms of survival 8. Perihepatic Metastases in advanced EOC patients occurs through the peritoneal spread of tumor implants on the liver surface. Sometimes, perihepatic metastases can invade the liver parenchymal 9. Several studies suggest surgery and platinum-based chemotherapy as the primary treatments associated with overall survival 10,11.

However, there are no LM screening guidelines for EOC patients with de novo LM. Hussain SM et al. stated that MRI is commonly used and considered optimal diagnostic modality for the assessment of alleged hepatic metastases 12. Other diagnostic modalities that often used are CT and PET-CT. Still, it could not identify the metastatic tumors less than 1.0 cm. also MRI, was not recommended for ovarian cancer patients' routine assessment in current guidelines for EOC patients screening 13,14.

Clinico-demographic characteristics of EOCLM patients, in the early estimation of the prognostic factors associated with LM can help the physicians to develop treatment strategies. Identification of potential risk factors and the importance of different treatment plans on EOCLM need to be assessed to provide alternative care and guidelines for the treatment of EOCLM patients based on a large population. Nevertheless, some studies with a minimal number of ovarian cancer patients with liver metastases were published before.

The Surveillance, Epidemiology, and End Results (SEER) is a publicly available cancer database that covers 30% of the United States population. The SEER system routinely records data on patient demographics, tumor characteristics, tumor morphology and histology, general treatment, stage at diagnosis, survival time, and follow-up. The current study aimed to identify the risk factors associated with de novo LM in EOC and to inspect the prevalence and survival trends of EOCLM patients. The study covers patients between the years 2010 and 2016 since the details of Liver Metastases (LM) were not available before 2010.

## Materials and Methods

### Study Population

Primary EOC patients diagnosed between the years 2010 and 2016 were identified using the National Cancer Institute's Surveillance, Epidemiology, and End Results (SEER) database 34. Patients are meeting the following criteria: The site-recode ICD-O-3(International Classification of Disease for Oncology-3)/WHO 2008^15^, primary site C56.9 restricted as “Ovary.” Patients diagnosed between 2010 and 2016, aged ≥18 years old; diagnosed with histologically confirmed invasive epithelial tumors and demographics (age, marital status, and race), clinicopathological parameters (stage, tumor grade, laterality, treatment details, survival and cause of death) included in the study cohort.

**Figure 1:**
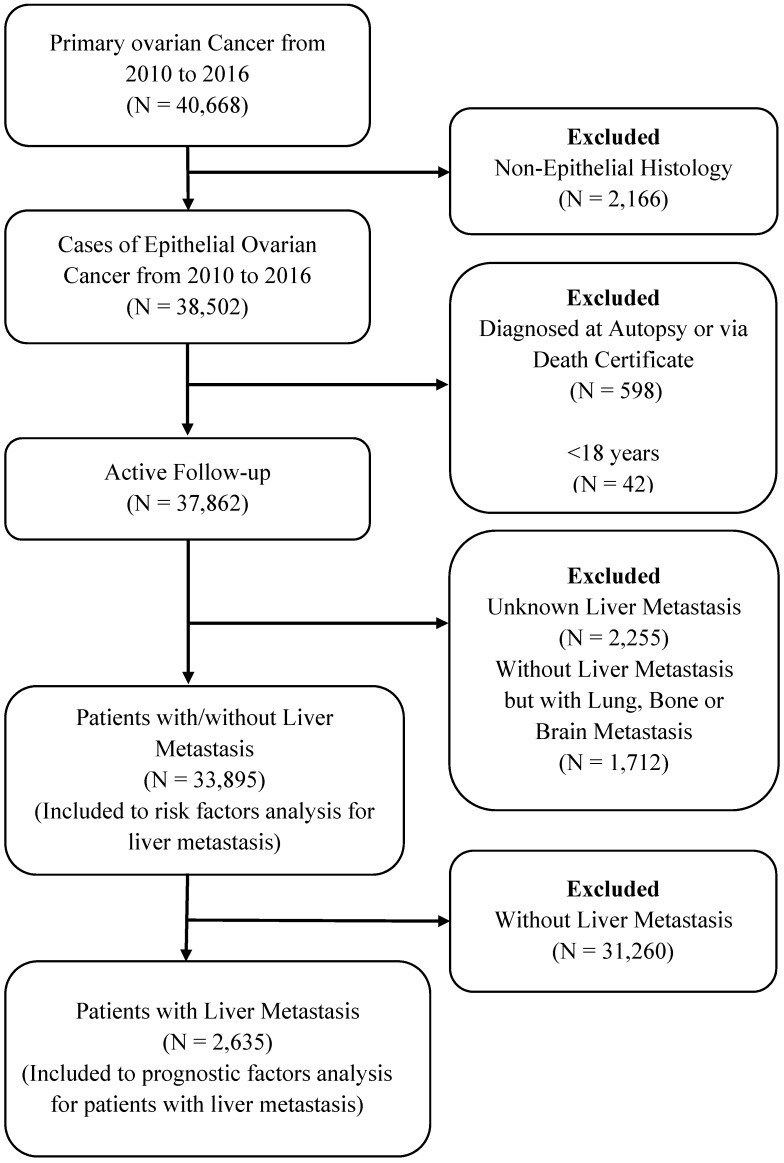
Flow-chart for patient selection in the present study.

Patients diagnosed by death certificate or autopsy only, patients with unknown information of follow-up or LM and patients without LM but lung, Bone, and Brain metastases were excluded from the study cohort. The detailed selection procedure is summarized in Figure 1. All data were extracted using SEER.Stat 8.3.5 software.

### Statistical Analysis

Demographic variables including age, race, marital status, and insurance were classified as followed: Age (≤40, 41-64 and ≥65 years), race (white, black and others), marital status (married and unmarried), and insurance status (insured, uninsured). Clinical variables, tumor location (one side, paired side and bilateral), cancer antigen-125 (normal and elevated), Tumor grade (I, II, III and IV), Tumor stage (T1, T2, and T3); regional lymph node (N0 and N1) defined according to the American Joint Committee on Cancer seventh edition (AJCC) 16. Histology was characterized as serous, endometrioid, mucinous, clear cell, Malignant Brenner Carcinoma NOS, Carcinosarcoma, and others 15,17.

Categorical variables are reported as counts (percentages). The chi-square test was used to analyze the differences between the subgroups. A *P*-value of less than 0.05 was considered statically significant. Univariable logistic regression was employed to predict potential risk factors for EOC patients with de novo LM. The factors statistically significant at the significance level of <0.05 were adjusted for further analysis using multivariable logistic regression.

The primary endpoint of the study cohort was to determine the overall survival of EOC patients with LM. A patient's survival was defined as the date of diagnosis until death from any cause. Kaplan-Meier curve method was employed to estimate overall survival, and the log-rank test was applied to evaluate the survival differences. Univariable Cox analysis was performed to investigate the prognostic factors for LM. Factors statistically significant at (p<0.05), in Univariable Cox Regression analysis, were then further evaluated using the Multivariable Cox model to classify the prognostic factors associated with overall survival among EOC patients with LM. All statistical analyses are performed using R version R-3.6.1 and Kaplan Meier curves were drawn using the R package ggplot2 18.

## Results

### Demographic and Clinical Characteristics

Based on selection criteria, a total of 33895 women diagnosed with EOC were involved in the retrospective study cohort (Figure 1). The average age of the women in the entire group was 62.57±14.21 years. Most patients were aged 40 and above years (94.24%), and 81.97% were white. Among these patients, 2,635 patients (7.77%) with EOC and LM had mean survival (23.73±24.27 months), and the mean age was 66.03±13.64 years. Detailed characteristics of the subjects are shown in Table 1.

### The Incidence of Liver Metastases (LM)

A total of 2635 primary EOC patients with distant liver Metastases (LM) were diagnosed between the years 2010 and 2016. Univariate analysis of subgroups showed patients ≥65 years of age (9.65%) presented with a significantly higher incidence of liver Metastases when compared with patients in lower age groups (χ^2^=145.55, p<0.001). Furthermore, black (12.17%) patients compared with white and others (χ^2^= 87.96, p<0.001), unmarried status (8.78%) (χ^2^=42.83, p<0.001), paired side laterality (16.87%) (χ^2^=734.36, p<0.001), Brenner tumor histology (17.63%) (χ^2^=939.44, p<0.001), higher T-stage (9.65%) (χ^2^=1405.20, p<0.001), N stage (12.14%) (χ^2^=428.72, p<0.001), poorly differentiated grade III (6.15%) (χ^2^=819.01, p<0.001), elevated cancer antigen-125 (8.61%) (χ^2^=150.60, p<0.001), surgery status “not performed” (19.91%) (χ^2^=1792, p<0.001), patients without chemotherapy (8.76%) (χ^2^=19.40, p<0.001) and patients who received radiotherapy (12.56%) (χ^2^=12.47, p<0.001) showed higher liver Metastases incidence than others. However, the incidence of liver Metastases showed no significant difference in insurance status (χ^2^= 3.94, p=0.139); (Table 1).

### Associated Risk Factors for Developing LM

Univariable logistic regression (Table 2) indicated that older age patients ≥40 years, black ethnicity, unmarried status, paired and bilateral tumor location, Brenner tumor histology, higher T stages T2 and T3, N1 stage, higher grades II, III and IV, elevated CA-125, and receipt of radiotherapy were all positively associated with LM risk.

On multivariable analysis Brenner tumor histology (OR 1.94, 95%CI 1.39-2.71, P<0.001), higher T stages T2/T1 (OR 2.28, 95%CI 1.49-3.50, *P*<0.001) and T3/T1 (OR 5.65, 95%CI 3.87-8.24, *P*<0.001), N-Stage (OR 1.67, 95%CI 1.43-1.95, *P*<0.001), poorly differentiated grade III/I (OR 2.16, 95%CI 1.29-3.59, p<0.001), and undifferentiated; anaplastic grade IV/I (OR 2.04, 95%CI 1.22-3.43, *P*<0.001), and elevated cancer antigen-125 (OR 1.79, 95%CI 1.19-2.69, *P*<0.001) remained independent characteristics associated with de novo LM.

### Cox Proportional Hazards Regression Analysis

Variables race, marital status, tumor location, histology types, surgery, chemotherapy, and radiotherapy were considered as potential prognostic factors included in the univariate cox regression model for pre-assessment. Age, tumor grade, cancer antigen-125, T-stage, and N-stage were excluded secondary to a lack of independent association with clinical outcomes. The results of the final multivariable cox model are listed in Table 3.

**Figure 2:**
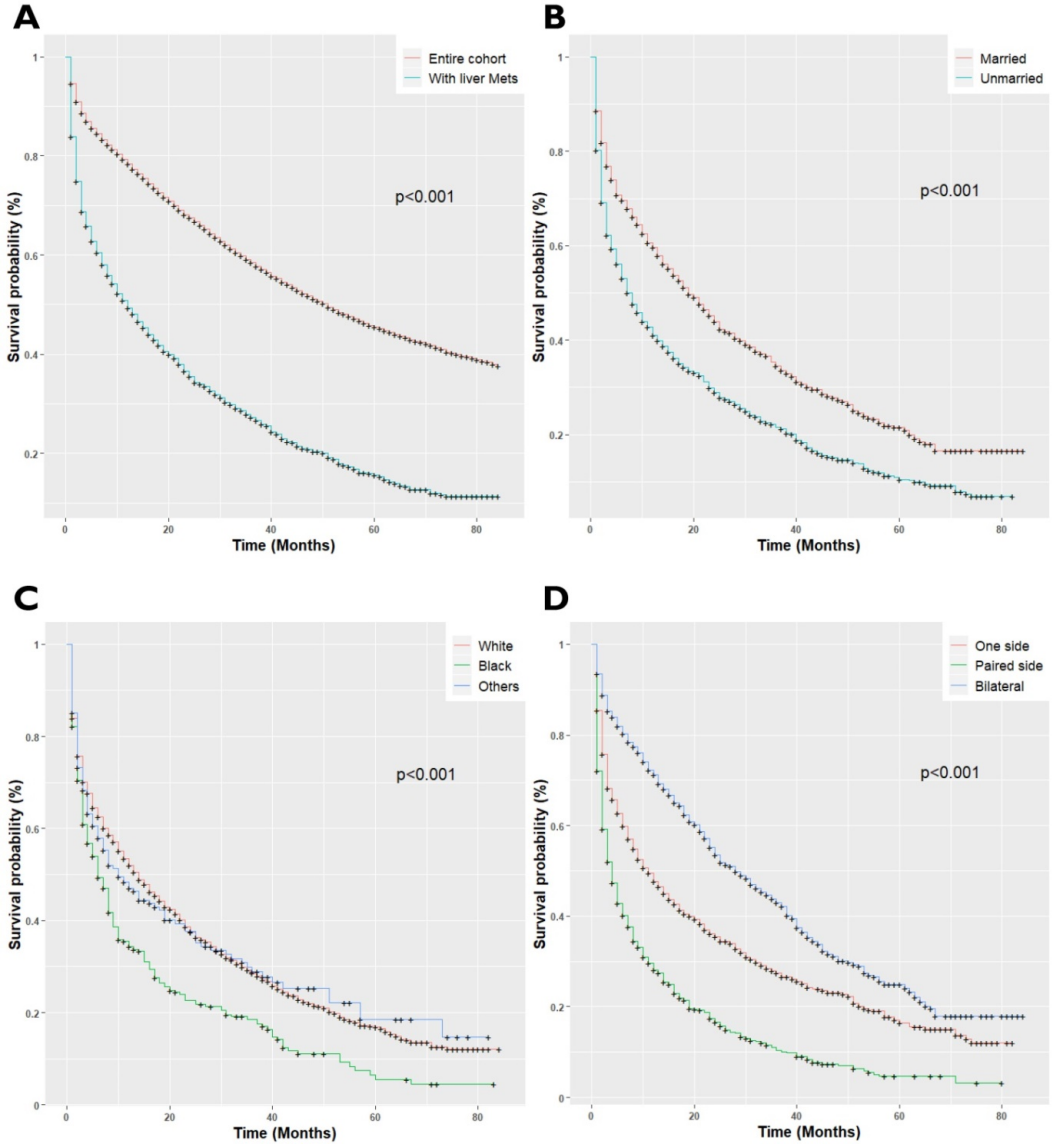

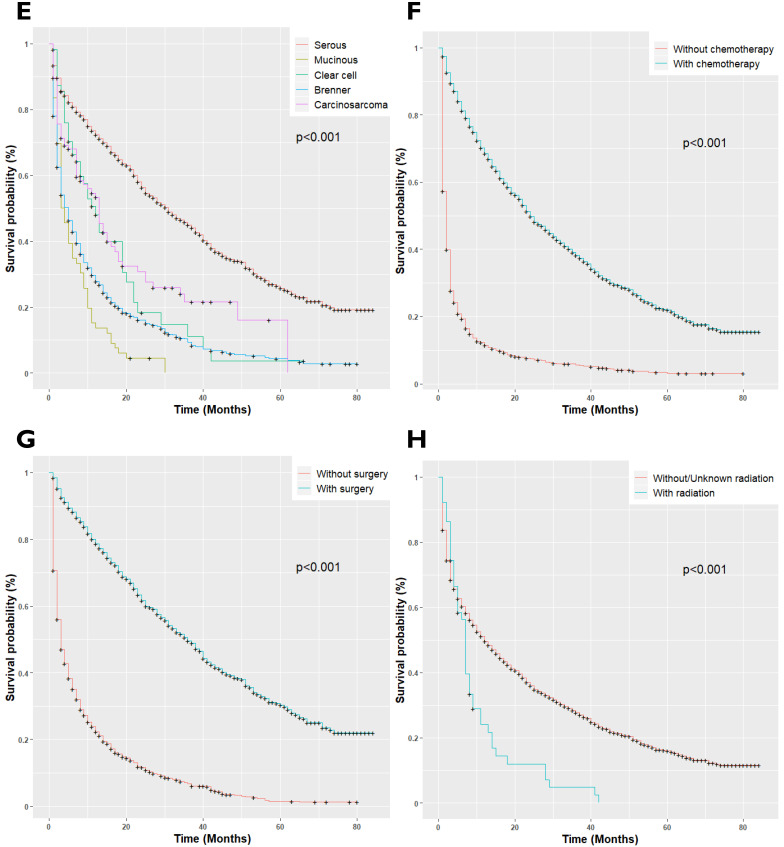
** (A)** Kaplan-Meier survival curves for patients with and without Liver metastases; **(B)** Kaplan-Meir survival curves for patients with Marital Status; **(C)** Kaplan-Meier survival curves for patients with race/ethnicity; **(D)** Kaplan-Meier survival curves for patients with tumor location; **(E)** Kaplan-Meier survival curves for patients with histology types; **(F)** Kaplan-Meier survival curves for patients with Neoadjuvant chemotherapy status; **(G)** Kaplan-Meier survival curves for patients with surgery status; **(H)** Kaplan-Meier survival curves for patients with radiotherapy status.

EOC patients with LM had a median survival of 12 months; 95%CI 11-14, *P*<0.001, as compared with the patients without LM (median survival 51.0 months, 95%CI 50-52, *P*<0.001). Once patients developed LM, the patient's survival rates plumped. The overall survival rates of EOC patients for 1-year and 3-year survival were 78% and 62%, respectively, while survival rates decreased to 49% and 27% respectively after LM diagnosis (*P*<0.001, Figure 2A). Univariate cox regression analysis showed, unmarried (*P*<0.001, Figure 2B), black race (*P*<0.001, Figure 2C), paired site tumor location (*P*<0.001, Figure 2D), non-serous histology types (*P*<0.001, Figure 2E), and receipt of radiotherapy (*P*<0.001, Figure 2H) was adversely associated with overall survival. Conversely, chemotherapy (*P*<0.001, Figure 2F), and surgical treatment of the primary site (*P*<0.001, Figure 2G) were positively associated with improved overall survival (*P*<0.001). The final Cox Model included independent prognostic factors with adjusted subgroups showed, when patients with married status were defined as the referent, the Hazards Ratio (HR) for disease progression in unmarried women was 1.16 (95%CI 1.04-1.30, *P*<0.001), with median survival and interquartile range (median 7.0; IQR 2.0-30.0) months. Those with non-serous histology types, the mucinous (HR: 2.38; 95%CI 1.82-3.12, *P*<0.001), clear cell (HR: 1.83; 95%CI: 1.32-2.55, *P*<0.001), Malignant Brenner Carcinoma, NOS, (HR: 1.44; 95%CI 1.26-1.66, *P*<0.001) and Carcinosarcoma (HR: 1.44; 95%CI 1.11-1.88, *P*<0.001) had a higher risk of disease prognosis. Receipt of radiotherapy was positively related to increased risk of death (HR: 1.52; 95%CI 1.12-2.06, *P*<0.001). Besides, patients who received Neoadjuvant chemotherapy (HR: 0.30; 95%CI 0.27-0.34, *P*<0.001) and with surgical treatment of the primary site (HR: 0.34; 95%CI 0.29-039, *P*<0.001), presented longer overall survival than those who did not receive treatment of chemotherapy. The median survival of the patients who received chemotherapy (Median survival: 24.0; IQR 9.0-54.0 months) and with surgery (Median survival: 36.0 IQR 15.0-71.0 months) as compared to the patients who did not receive chemotherapy (Median survival: 2.0; IQR 1.0-4.0 months) and surgery (Median survival: 3.0; IQR 1.0-11.0 months) respectively.

## Discussion

To our knowledge, this is the first large population-based study to explore the demographic and clinical characteristics, potential risks, and prognostic factors of LM from EOC that have been analyzed premortem up to this point. Based on our retrospective cohort, 7.77% of primary EOC patients initially diagnosed with de novo LM. Winter et al. reported that Hepatic parenchymal Metastases accounted for 18% and was the second most common cause of stage-IV disease in a large GOG study 19. The metastatic rate to the liver in our study was lower than the previous studies, as most of the studies restricted to the distant stage (stage-IV) disease. The prognostic role of the LM pattern in EOC patients remains to be explained. Potential risk factors identification is needed to investigate for LM progression and screening performance among high-risk EOC patients.

The current study identified older age, black race, unmarried status, paired site tumor location, Brenner tumor histology type, higher T-stage, N stage, poorly differentiated grades III and IV, cancer antigen-125, and receipt of radiotherapy was significantly associated with de novo LM development. Our findings corroborate the findings of previous studies 20-23. Mizuno et al. found by analyzing 223 EOC patients that non-serous tumor histology types, the clear cell and mucinous were significantly associated with worse prognosis and a higher rate of early deaths compared to the Serous and endometrioid histology tumors (*P*<0.001) 24. The results of the current study provide further support to the evidence suggesting non-serous histology types have reduced survival, with the median overall survival of 4-13 months as compared to 31-34 months in serous and endometrioid histology types 25.

In our study, EOCLM patients who underwent primary surgery had higher survival compared to those who did not experience this procedure. Almost 47% of the patients underwent surgical cytoreduction of the primary site; the data still suggest that the surgical approach of primary cytoreduction in advanced-stage disease is interrelated with improved overall survival of EOCLM patients. Winter et al. observed a prolonged survival in EOCLM patients who received primary cytoreductive surgery 19. Additionally, prior studies also suggested that cytoreductive surgery of primary site of EOC patients with FIGO stage-IV disease more likely to have immense advantages 10,26,27.

Chemotherapy is commonly employed in patients with advanced-stage EOC patients. 66.33% of patients in our study received chemotherapy. Our results suggest that chemotherapy offers enormous benefits in the adjuvant treatment of women with EOCLM. In the current analysis, it was observed that chemotherapy admiration was associated with highly improved survival. It is prominent since this strengthens the reason for better assumptions for median progression-free and overall survival. The study findings are similar to those in other regional or multicenter studies that analyzed distant-stage epithelial ovarian carcinomas separately 28-33.

One of the main strengths of the current study is the fact that it presents a large number of LM patients from nationwide registries. Moreover, the study period spanned seven years with active follow-up. Conversely, several limitations should be noted. First of all, the SEER database does not provide information on the administration of hepatic resection during cytoreductive surgery for primary EOCLM patients. Comorbidity profiles of the patients, such as hepatic involvement, the liver parenchymal invasion, hematogenous dissemination, and patient's preference to receive primary or secondary metastatic surgery, which may partially affect the precision of the results. Secondly, our results showed a strong association between the surgery of the primary site and improvement in survival among EOCLM patients. However, the information regarding the history of the disease, and treatment morbidity is lacking. Thirdly, the SEER database does not provide information regarding those EOC patients who have developed liver metastases later during follow-up. Lastly, one of the limitations of the current research was the lack of validation of the present study through an independent cohort as actively followed EOC patients with LM lacked in other databases. Therefore, the observed LM of the current research has to be interpreted as an underestimate of the real figure.

In conclusion, our study clarified the epidemiology of EOCLM in US women. The survival of women with liver Metastases who underwent surgery of primary site and chemotherapy exceeds that of patients without surgery or chemotherapy. Besides, radiation therapy was negatively associated with the overall survival of the patients. The prognosis of EOCLM patients is poor, with a median survival of 12 months. Non-serous histology-types, the mucinous, clear cell, Malignant Brenner Carcinoma, NOS, and Carcinosarcoma, were positively associated with overall death. Comparatively, good survival was observed in the white race, married women, and those who had one-side or bilateral tumors. The limitations of the present study support the need for related studies to further confirm the results in the future.

## Figures and Tables

**Table 1:** Demographic and clinical characteristics for epithelial ovarian cancer patients diagnosed with and without liver metastases (LM)

Subject characteristic	No. of epithelial ovarian cancer patients		χ^2^	*P* value^a^
	With LM	Without LM		
	(N=2635, 7.77%)	(N=31260, 92.23%)		
**Age, in years**	66.03±13.64	62.28±14.22	145.55	<0.001
18-40	87(4.45)	1867(95.55)		
41-64	1079(6.45)	15641(93.55)		
≥ 65	1469(9.65)	13752(90.35)		
**Race**			87.96	<0.001
White	2080(7.49)	25703(92.51)		
Black	341(12.17)	2461(87.83)		
Others	208(6.58)	2952(93.42)		
Unknown	6(4.00)	144(96.00)		
**Marital status**			42.83	<0.001
Married	1124(6.83)	15330(93.17)		
Unmarried	1395(8.78)	14500(91.22)		
Unknown	116(7.50)	1430(92.50)		
**Insurance**			3.94	0.139
Insured	2481(7.71)	29699(92.29)		
Uninsured	107(9.22)	1054(90.78)		
Unknown	47(8.48)	507(91.52)		
**Tumor Location**			734.36	<0.001
One side	931(5.19)	16998(94.81)		
Paired side	830(16.87)	4091(83.13)		
Bilateral	874(7.91)	10171(92.09)		
**Histology**			939.44	<0.001
Serous	1200(7.02)	15905(92.98)		
Endometrioid	65(1.78)	3579(98.22)		
Mucinous	73(3.63)	1940(96.37)		
Clear cell	56(2.67)	2044(97.33)		
Brenner	581(17.63)	2714(82.37)		
Carcinosarcoma	97(8.27)	1076(91.73)		
Others	399(11.06)	3210(88.94)		
Unknown	164(17.15)	792(82.85)		
**T stage**			1405.20	<0.001
T1	119(1.34)	8747(98.66)		
T2	200(4.40)	4345(95.60)		
T3	1692(9.65)	15840(90.35)		
Unknown	624(21.14)	2328(78.86)		
**N stage**			967.22	<0.001
N0	1182(5.00)	22459(95.00)		
N1	852(12.14)	6168(87.86)		
Unknown	601(18.58)	2633(81.42)		
**Grade**			819.01	<0.001
I	27(1.06)	2509(98.94)		
II	94(2.63)	3485(97.37)		
III	615(6.66)	8617(93.34)		
IV	402(5.59)	6792(94.41)		
Unknown	1497(13.18)	9857(86.82)		
**CA-125^b^**			150.60	<0.001
Normal	62(2.17)	2794(97.83)		
Elevated	2022(8.61)	21455(91.39)		
Unknown	551(7.29)	7011(92.71)		
**Surgery**			1792.70	<0.001
None	1370(19.91)	5510(80.09)		
Yes	1236(4.62)	25540(95.38)		
Unknown	29(12.13)	210(87.87)		
**Chemotherapy**			19.40	<0.001
None/Unknown	887(8.76)	9238(91.24)		
Yes	1748(7.35)	22022(92.65)		
**Radiation**			12.47	<0.001
None/Unknown	2584(7.72)	30905(92.28)		
Yes	51(12.56)	355(87.44)		

^a^ P-value for the χ^2^ test for the distribution of categorical variables by liver metastases (LM); ^b^ CA-125: Cancer Antigen 125.

**Table 2:** Univariable and Multivariable Logistic Regression analysis of EOC patients with Liver Metastases

Subject characteristics	Univariable		Multivariable	
	OR (95%CI)	*P*-value	OR (95%CI)	*P*-value
**Age, in years**				
18-40	Ref	1.00	Ref	1.00
41-64	1.48(1.18-1.85)	**<0.001**	0.80(0.55-1.16)	0.234
≥ 65	2.29(1.84-2.86)	**<0.001**	0.83(0.57-1.21)	0.335
**Race**				
White	Ref	1.00	Ref	1.00
Black	1.71(1.52-1.93)	**<0.001**	1.16(0.88-1.53)	0.27
Others	0.87(0.75-1.00)	0.111	1.04(0.80-1.35)	0.75
Unknown	NA	NA	NA	NA
**Marital status**				
Married	Ref	1.00	Ref	1.00
Unmarried	1.31(1.21-1.42)	**<0.001**	1.14(0.98-1.33)	0.090
Unknown	NA	NA	NA	NA
**Insurance**				
Insured	Ref	1.00	Ref	1.00
Uninsured	1.22(0.99-1.49)	0.059	0.90(0.57-1.43)	0.658
Unknown	NA	NA	NA	NA
**Tumor Location**				
One side	Ref	1.00	Ref	1.00
Paired side	3.70(3.35-4.09)	**<0.001**	1.29(0.92-1.81)	0.139
Bilateral	1.57(1.43-1.73)	**<0.001**	1.04(0.88-1.22)	0.644
**Histology**				
Serous	Ref	1.00	Ref	1.00
Endometrioid	0.24(0.19-0.31)	**<0.001**	1.03(0.71-1.49)	0.862
Mucinous	0.49(0.39-0.63)	**<0.001**	1.22(0.71-2.12)	0.462
Clear cell	0.36(0.28-0.48)	**<0.001**	0.72(0.43-1.19)	0.194
Brenner	2.83(2.54-3.16)	**<0.001**	1.94(1.39-2.71	**<0.001**
Carcinosarcoma	1.19(0.96-1.48)	0.106	1.06(0.72-1.57)	0.766
Others	1.64(1.46-1.86)	**<0.001**	1.28(0.99-1.66)	0.057
Unknown	NA	NA	NA	NA
**T stage**				
T1	Ref	1.00	Ref	1.00
T2	3.38(2.69-4.26)	**<0.001**	2.28(1.49-3.50)	**<0.001**
T3	7.85(6.51-9.47)	**<0.001**	5.65(3.87-8.24)	**<0.001**
Unknown	NA	NA	NA	NA
**N stage**				
N0	Ref	1.00	Ref	1.00
N1	2.63(2.39-2.87)	**<0.001**	1.67(1.43-1.95)	**<0.001**
Unknown	NA	NA	NA	NA
**Grade**				
I	Ref	1.00	Ref	1.00
II	2.51(1.63-3.86)	**<0.001**	1.42(0.83-2.45)	0.201
III	6.63(4.50-9.78)	**<0.001**	2.16(1.29-3.59)	**0.003**
IV	5.50(3.72-8.14)	**<0.001**	2.04(1.22-3.43)	**0.006**
Unknown	NA	NA	NA	NA
**CA-125**				
Normal	Ref	1.00	Ref	1.00
Elevated	4.25(3.29-5.48)	**<0.001**	1.79(1.19-2.69)	**<0.001**
Unknown	NA	NA	NA	NA
**Surgery**				
None	Ref	1.00	Ref	1.00
Yes	0.19(0.18-0.21)	**<0.001**	0.32(0.24-0.34)	**<0.001**
Unknown	NA	NA	NA	NA
**Chemotherapy**				
No/Unknown	Ref	1.00	Ref	1.00
Yes	0.83(0.76-0.90)	**<0.001**	1.096(0.87-1.377)	0.430
**Radiation**				
None/Unknown	Ref	1.00	Ref	1.00
Yes	1.71(1.28-2.31)	**<0.001**	1.58(0.89-2.80)	0.120

Abbreviations: CA-125: cancer antigen 125; NA: Not available; Factors with Unknown data were adjusted for the logistic regression model.

**Table 3:** Univariable and Multivariable Cox Regression for analyzing the prognosis factors for epithelial ovarian cancer with liver metastases

Subject characteristics	Survival month, Median(IQR)	Univariable		Multivariable	
		HR (95%CI)	*P*-value	HR (95%CI)	*P*-value
**Race**					
White	14(3.0-42.0)	Ref	1	Ref	1
Black	6(2.0-20.0)	1.47(1.29-1.67)	**<0.001**	1.14(0.99-1.32)	0.064
Others	10(2.0-51.0)	1.02(0.68-1.22)	0.794	1.17(0.96-1.41)	0.114
**Marital status**					
Married	19(4.0-52.0)	Ref	1	Ref	1
Unmarried	7(2.0-30.0)	1.55(1.41-1.71)	**<0.001**	1.16(1.04-1.30)	**0.007**
**Tumor Location**					
One side	11(3.0-42.0)	Ref	1	Ref	1
Paired side	4(1.0-15.0)	1.83(1.64-2.04)	**<0.001**	0.99(0.88-1.12)	0.902
Bilateral	28(10.0-57.0)	0.63(0.56-0.71)	**<0.001**	0.93(0.83-1.07)	0.33
**Histology**					
Serous	31(10.0-62.0)	Ref	1	Ref	1
Endometrioid	34(8.0-65.0)	0.92(0.65-1.31)	0.654	0.87(0.60-1.24)	0.438
Mucinous	4(2.0-10.0)	4.17(3.22-5.39)	**<0.001**	2.38(1.82-3.12)	**<0.001**
Clear cell	12(5.0-22.0)	2.04(1.48-2.80)	**<0.001**	1.83(1.32-2.55)	**<0.001**
Brenner	5(2.0-14.0)	3.13(2.78-3.53)	**<0.001**	1.44(1.26-1.66)	**<0.001**
Carcinosarcoma	13(3.0-34.0)	1.87(1.45-2.41)	**<0.001**	1.44(1.11-1.88)	**<0.001**
Others	4(1.0-19.0)	2.80(2.45-3.20)	**<0.001**	1.47(1.27-1.70)	**<0.001**
**Surgery (Pri.)**					
None	3(1.0-11.0)	Ref	1	Ref	1
Yes	36(15.0-71.0)	0.21(0.19-0.23)	**<0.001**	0.34(0.29-0.39)	**<0.001**
**Chemotherapy**					
None/Unknown	2(1.0-4.0)	Ref	1	Ref	1
Yes	24(9.0-54.0)	0.20(0.18-0.22)	**<0.001**	0.30(0.27-0.34)	**<0.001**
**Radiation**					
None/Unknown	12(2.0-40.0)	Ref	1	Ref	1
Yes	7(3.0-11.0)	1.74(1.30-2.33)	**<0.001**	1.52(1.12-2.04)	**<0.001**

Abbreviations: IQR: interquartile range; Surgery (Pri.): surgery on primary site; The above factors with Unknown data were removed from Kaplan-Meier and Cox regression models.
